# The Future of Morphological Science Education: Learning and Teaching Anatomy in the Wake of the COVID-19 Pandemic

**DOI:** 10.3390/ijerph20075367

**Published:** 2023-04-03

**Authors:** Amr Maani, Alicja Forma, Adam Brachet, Katarzyna Czarnek, Abduelmenem Alashkham, Jacek Baj

**Affiliations:** 1 Jackson Park Hospital & Medical Center, 7531 Stony Island Ave, Chicago, IL 60649, USA; 2 Department of Anatomy, Medical University of Lublin, 20-090 Lublin, Poland; 3 Department of Forensic Medicine, Medical University of Lublin, 20-059 Lublin, Poland; 4 Institute of Health Sciences, Faculty of Science and Health Sciences in Lublin, The John Paul II Catholic University of Lublin, ul. Konstantynów 1 H, 20-708 Lublin, Poland; 5 Anatomy, School of Biomedical Sciences: Edinburgh Medical School, University of Edinburgh, Edinburgh EH8 9AG, UK

**Keywords:** morphological sciences, medical education, anatomical education, distance learning, COVID-19 pandemic

## Abstract

The COVID-19 Pandemic has conveyed an unprecedented worldwide challenge. Although there is much emphasis on caring for patients and communities, the high incidence of SARS-CoV-2 had seriously disturbed education and calls for prompt as well as serious consideration from educators in medical schools. The necessity to teach and prepare prospective medics, as well as clinicians, has certainly not been as intense as it is currently. The global effects of coronavirus disease 2019 may cause a permanent change in the education of future clinicians. The COVID-19 era presented logistical and practical obstacles and fears for the patients’ well-being, taking into consideration the fact that students may be potential channels for the spread of the virus when asymptomatic and may get infected while being in training and attending lectures. This paper discusses the present state of morphological science education, depicting the effect of COVID-19 on learning environments, as well as highlights the probable effects of COVID-19 on medical instruction in the future.

## 1. Medical Education in the 21st Century

For over a decade, medical schools have been constantly changing their pedagogy by means of decreasing the number of lectures as well as replacing laboratory sessions with new innovative technology, implementing self-directed, active, team-coordinated learning as well as self-education, and promoting interprofessional development [1,2]. Assessment has been transformed through the development of competency-based learning and professional activities with recognized milestones for achievement. The majority of medical universities have shortened the duration of basic science curricula to at most two years or a year-and-a-half. They have also integrated clinical education into this time frame while retouching upon basic medical science as students advance in their studies [3].

Presently, within many medical universities, the student convenes physically in the initial two years for discussions or problem solving within a group, and it is worth noting that the student presence in both inpatient, as well as outpatient, environments has been a major component of the early clinical immersion experience. The final two years of medical education are usually clinical. Medical students take part in rotations, clerkships, and internships just before their specialty. It is a fact that COVID-19 may affect students all throughout the educational process.

## 2. Teaching Practices in Anatomy Education

Human anatomy is recognized as one of the main cornerstones in medicine, and it is often the foundation upon which clinicians develop their skills [4,5,6]. For this reason, it is imperative that a deep and thorough understanding of anatomy is acquired, thus resulting in safe practice in the clinical setting, specifically in the area of surgery [7,8]. Teaching is a process that undergoes constant evolution, adjusting to various external factors, producing revised approaches that improve the learning method; this is also factual for teaching anatomy [9,10,11,12].

An anatomist makes a good doctor. Suffice it to say that anatomy is the basis of the medical curricula, and a good physician improves their skills based on this. Anatomical knowledge is necessary for physicians to practice safely, especially with respect to surgery [13]. To teach anatomy effectively, the teacher must review their teaching approach constantly [9]. Constant analysis is also important, as it helps to regulate the best approaches as well as tools that best suit the education activity [9]. Recently, we have witnessed a decrease in traditional cadaveric-based teaching, which is triggered by a shift towards a system-based or integrated curriculum [14,15]. Cost factors, religious beliefs, and time factors also contribute immensely to this reduction. Additionally, it is of note that not much time is allotted to anatomy teaching in our medical institutions [14,16].

Medico-legal litigation for malpractice in surgery has steadily been on the rise [17]. In the UK, in the years 1995–2000, there has been a rise in the total number of claims bordering on anatomy incompetence, all submitted to the Medical Union Defense. According to the report [18], 32 percent of the claims were made against vascular and general surgeons, all alleging “damage to underlying structures.” A study conducted by Cahill et al. [19] presented that a significant percentage of the over 80,000 yearly preventable deaths within the United Kingdom could be attributed to anatomy mistakes and incompetence on the part of the clinicians [19]. It has been reported that less than a third of new surgical trainees had a sound understanding of human anatomy [20], but despite this, many medical schools drastically reduce the teaching time allotted to anatomy, thus enhancing the drop in anatomical knowledge among medical and dental students (both undergraduate and postgraduate) [9,21]. Most of the previous publications have focused more on anatomy curricula, compared to other medical disciplines (pharmacology, biochemistry, biology, or physiology) [22].

Brenner et al. [23] classified the teaching tools into six categories, namely: first, dissection handled by medical students; second, prosection inspection; third, didactic instruction; fourth, the usage of models; fifth, technology-based learning such as computer-based; and sixth, the usage of radiological as well as living examples in teaching anatomy [15]. Considering the changes that have been effected in the anatomy curriculum, many studies have explored the attitude of anatomy teachers and students towards the modalities of teaching [9,24,25].

### 2.1. Cadaver Dissection

Traditional teaching methods often involve the use of a blackboard and chalk as the main tool of information delivery. However, dissection of the human body has also remained the main teaching tool in anatomy education for over 400 years [25,26]. However, over the last few years, there has been a decrease in the number of hours that are assigned to cadaveric dissection in the curriculum due to the lack of availability of cadavers and an increase in costs of the running of dissection in laboratories [27].

Studying with human cadavers has innumerable advantages, including enhancement of effective learning as well as preparing students for their clinical training, preparation for death news, mastering manual skills, and having a firm knowledge of the association between symptoms experienced by the patient and the pathology [25,28]. It also develops medical professionalism, such as empathy, strategies for coping with stress, and teamwork competency [29].

Compared to plastination, teaching with cadavers and doing dissections provide the much-needed element of surprise when the student identifies an anatomical variant [30], shows finer details, and maintains the texture close to that of a living body, giving the students the feeling of being in an operating theatre [31]. Without a doubt, the dissection of the cadaver plays a very important role in anatomy education as well as the training of future doctors [32]. In addition, it can also aid in the improvement in teamwork, medical professionalism, empathy, and ability to experience stress-handling mechanisms [29].

### 2.2. Prosections

A cadaveric prosection is defined as a previously dissected specimen, which is occasionally plastinated [33]. Prosections have been a key part of anatomical teaching since the Middle Ages as well as the early Renaissance period [34,35]. With reduced teaching time for anatomy in an integrated curriculum as well as a shortfall in the number of bodies donated, many programs have resorted to prosections, thus bringing down the amount of interaction time, while still exposing students to the anatomical material that they may otherwise waste an inordinate amount of time trying to locate [36]. For example, structures such as the major vessels below the heart lie deep in the posterior abdominal region.

Dissecting these structures according to their region is a time-consuming task. Thus, dissection does not fit into a system-based approach; learning anatomy seems better through dissections compared to the regional dissecting technique [37]. A study has shown that despite the increasing number of cadavers, no anatomy program within New Zealand and Australian Universities is wholly dissection-based [5]. According to a survey of New Zealand and Australian medical schools, the learning curriculum applied for teaching anatomy is case-based, integrated and systemic [5].

The advantages offered by prosection include flexibility, time-efficiency and context, considering that it is easy to observe the structures and their relations, and not many cadavers are needed, as the prosections can be utilized by more than one student cohort [35,38,39]. By studying from prosected specimens, medical students are imbued with the ability to identify anatomical variants in many specimens, compared to those who dissect cadavers [40].

### 2.3. Computer-Based Learning

With recent technological advances, high costs of cadaver-based teaching, increase in class size, and reduced teaching time, resources for computer-based learning are used more in anatomy teaching to help the student understand the subject [25,40]. The use of computer-based learning augments the teaching of anatomy, encourages independent learning, provides flexibility, and enhances problem-solving [41]. We do not have conclusive proof that computer-based learning has more advantages than conventional teaching methods [42,43], but then, studies suggest that computer-based learning of anatomy enhances learning through supplementation, compared to conventional teaching methods [40,41]. Many students prefer conventional approaches such as prosection, dissection, and textbooks, as well as lectures conducted via computer-based learning resources [25]. In the same way, anatomists share the opinion that cadaveric teaching is of course mandatory for ideal education, even if used alongside computer-based learning resources such as the 3D technology and medical simulations [5,40].

The advancement of digital medical technology provided the ability to generate two-dimensional images from cat scans and MRIs. These images are now quite rapidly transformed into three-dimensional images to aid in disease diagnoses [44,45]. Three-dimensional learning applications are an efficient learning method for studying gross anatomy, since it allows students to identify 3D atlas applications, including 2D applications [46]. According to a recent study, anatomy 3D models are considered to be an efficient multimedia method for anatomy education [47]. Another aspect in 3D technology that is increasingly being considered in almost all fields of science and technology is 3D printing [48,49,50]. This is a prototyping technology that shows immense potential for anatomy teaching and learning. The principle regarding this innovative technology is the usage of computer-aided design for the rebuilding of a physical 3D model. From this data, the model is constructed from a sequence of cross-segments by use of the 3D printer, which lays down consecutive levels of liquid or powder as well as a plastic material [51]. Three-dimensional printing has the ability to reproduce high-precision models with lower cost as well as within a relatively short amount of time, greatly assisting students in understanding structures and relationships between structures [7].

In addition to 3D technology, simulators have been proven to be great tools that provide simulations, which are computer-generated images or animations that imitate the properties of a real object, and have become an important teaching tool since the 1990s [52,53]. Simulators are extremely useful in understanding some key concepts in anatomy and the function of the body [54,55]. In addition to this, they can also enhance theoretical teaching and practical skills [56]. It has further been reported that stimulation assists students to become more motivated learners and invest more of their resources of working memory, compared to those who are less motivated in dealing with complex instructions for better learning [57].

### 2.4. Peer Teaching and Learning in Anatomy

Peer teaching can be an effective method for teaching anatomy during the COVID-19 pandemic. This method involves students learning from each other, sharing knowledge and experience. As part of peer teaching, students can form groups where they discuss and explain difficult anatomical concepts to each other. They can also organize online meetings where they present their own materials and exercises and discuss them together. This allows students to exchange knowledge and skills, as well as to learn from other students who have different ways of learning [58]. An important aspect of peer teaching is self-assessment, which involves reflecting on one’s own skills and knowledge. Students should learn to identify their strengths and weaknesses and seek ways to improve them [59]. Peer teaching involves a student or learner teaching others in their group, thereby consolidating their own knowledge and skills [60]. For learning anatomy, peer teaching may involve creating discussion groups or online meetings where students share their knowledge and help each other understand difficult concepts. Peer teaching can be an effective way to learn teaching techniques. When you engage in peer teaching, you have the opportunity to practice your teaching skills in a safe and supportive environment. This can help you to build your confidence and develop new teaching techniques [61]. It is also important to note that peer teaching does not replace traditional teaching but, rather, complements it. Therefore, it is important for students to have access to appropriate educational materials, such as books and videos, and to have the opportunity to consult with teachers or tutors. Peer teaching has many benefits; it may not be the best approach for every situation or every learner. Educators should consider the potential drawbacks and make sure that peer teaching is appropriate for the context and the needs of the learners involved [62,63]. Summary:

- Peer teaching can be an effective method for teaching anatomy during the COVID-19 pandemic.
- Students can form groups, organize online meetings, present their own materials and exercises, and discuss them together.
- Self-assessment is important, involving reflecting on one’s own skills and knowledge.
- Peer teaching complements traditional learning, so it is important for students to have access to appropriate educational materials and to have the opportunity to consult with teachers or tutors.

## 3. Medical Education during COVID-19

As nations strive to control the rapid spread of COVID-19, and with almost the majority of the global population being locked down [64], the impact that this pandemic has had on the lives of people is far above whatever experience people may have had in the past. The education sector is one of the most affected [65]. In the UK and US for instance, most medical schools and universities had suspended physical classes by March 2020 [66], thus compelling students to switch to online distance learning. Moszkowicz et al. [67] proposed the videoconferencing solution for universities in the United States. Videoconferencing is a relatively newer teaching technology that has been used in many teaching environments [68]. This approach is believed to some extent to be sufficient for clinical training carried out on a day-to-day basis in the surgery department. In addition to this, this method has also been successfully used in performing real life surgeries using wireless remote control video conferencing equipment [69]. Both anatomy and clinical lessons will benefit from the videoconferencing solution.

Moszkowicz et al. [67] used the Google Hangouts app, which is totally free and easily accessed in the Gmail application list. Moreover, a recent study investigated how videoconferencing can enable clinical expertise to be delivered to an institution during a training session and to further provide national and international experts for presentations and teaching [70]. The gathered student feedback was extremely encouraging regarding the session, which was rated as ‘outstanding’. It was also a success owing to the presence of both teachers and patients as well as the possibility of having a discussion with experts in the field. One of the major advantages of this method is the real-time teaching delivery during remote student clinical placement. These types of conference tools could strengthen the significance of anatomy, underpinning the medical clinical practice. Videoconferencing also has its drawbacks, and some of these include (1) the lack of a whiteboard tool on the videoconferencing application, and more importantly, (2) the need and availability for high-speed uninterrupted Internet connectivity [70].

The faculties of medicine and science in most Australian and New Zealand Universities have switched to remote and/or online delivery as staff has reverted to a new routine of functioning from home. According to Simonson et al. [71], remote learning means a formal, and university-based learning, in which the instructor as well as the student are physically disconnected but connect to each other by using interactive telecommunication systems. Australia has not left any stone unturned, owing to its pragmatism to boost their healthcare system by 2021, with heavy reliance upon the graduation of medical students into the health system as junior physicians on a yearly basis.

For anatomy as a discipline, this particular choice has some significance. The COVID-19 pandemic reaction, as mandated by the government, resulted in physical distancing and movement restriction. At first, these regulations constrained access to anatomy laboratories but progressed quickly to a prohibition of student access to the labs, as learning via distance had become the new norm. The early phase of this change might be tough for medical students, as most traditional pedagogies depend solely on the hands-on practical sessions. In addition, many anatomy educators identify through these small groups the learning needs of the students, especially those who are close to underperforming [72]. Furthermore, an online delivery of anatomy lectures poses a great challenge to the long-held philosophy of anatomy teachers [38].

According to a SWOT analysis from Longhurst et al. [73], universities located within the United Kingdom as well as Ireland replaced lectures with recorded presentations provided to students found on the virtual learning environment (VLE). For example, the usage of Panopto was the highest. Over 50% of universities have acknowledged it. A total of 36% of universities taught via Zoom, Big Blue Button (Ottawa, Canada), and Collaborate Ultra (New York, NY, USA). Practical sessions in these institutions are supplemented with virtual learning environments with extra resources. Digitized cadaveric resources are used by only 29% of universities, while 7% use 3D virtual resources, and 43% combine both cadaveric dissection and 3D sources. Digitized human cadaveric dissection used includes Acland’s Video Atlas, YouTube videos, high-quality cadaveric images, the Human Visible project (US national Library of Medicine, Bethesda, MD, USA), and bespoke videos of prosected specimens [73,74].

## 4. The Effect of the COVID-19 Pandemic on Medical Education

COVID-19 is not the first pandemic to hit the world. Galen of Pergamon wrote about the smallpox pandemic that lasted 20 years. According to the report, the pandemic began in 166 AD [75]. In 2003, the world faced an acute severe respiratory syndrome [76] and then the 2009 H1N1 swine flu pandemic [77]. Without a doubt, COVID-19 may be the first to affect medical students. However, we can draw lessons we learned from the past crises we experienced—lessons that will aid us to adjust as well as to carry on with anatomical education. For instance, online education recorded high success when it was employed in the 2003 pandemic [78,79] and has once again been employed effectively [80]. Students can learn anatomy even without the use of a cadaver, entirely from online resources and textbooks [81], and, quite frankly, many medical schools have done away with cadaver dissection [82].

However, reduced practical sessions due to COVID-19 will have negative impacts on students, regardless of whether they work with cadavers or not. The current instructional setting is less suitable for learners, and the absence of physical interaction among teachers as well as peers could hinder the fast and smooth development of the student as an anatomist. Despite the oodles of technological advancements that enhance online distance learning, learning anatomy in the laboratory, mainly through the dissection of cadavers, is regarded as an effective learning approach [26]. Loss of access to dissection rooms also implies a loss of access to cadavers as well as a wide range of effective learning modalities such as models, skeletons, prosections, and pathological specimens [83]. Some researchers had stated that the 21st-century curriculum in medicine has placed a lot of restrictions on students’ exposure to anatomy [84], and the COVID-19 era has additionally reduced the amount of contact time received by learners. As such, medical students receive their lessons with little or no access to learning materials that are practical-based, such as prosections, models, or cadavers.

Teaching anatomy without the use of cadavers is a seemingly non-favorable practice, although it has some advantages, as well as having been employed in various universities [85]; learning becomes difficult when models, prosections, and other learning materials are excluded as well. Getting used to online learning may not be a simple procedure for teachers or students, and making an online atlas available might not help medical students “appreciate the human body” [86]. There is a great number of anatomy educational online software programs created for medical students, but they might be costly. Capable institutions should purchase and allow their students to access these software programs amid the pandemic and even beyond. However, in order not to create a disparity between institutions with varying financial capabilities, software makers must consider giving medical students trial access while the COVID pandemic lasts. That said, past research showed that, despite their usefulness, online software programs do not deliver satisfactorily high rates of satisfaction and self-perceived education as opposed to cadaveric dissection sessions [87]. Additionally, the use of these programs by teachers and students has been associated with a steep learning curve [88].

There are reports that a large number of medical students had a hard experience manipulating models as well as focusing on those structures in which they have an interest [89]. This brings their usefulness into question in perilous times such as a pandemic. However, no studies have been carried out to investigate the efficacy of online programs. It is a fact that the COVID-19 pandemic might aid in educating educators on if these methods have the potential to deliver adequate educational benefits, and also on whether the integration of the new technology into the medical curriculum and its usage to aid learners with care throughout their learning experience, instead of only serving as an alternative source, might lead to an efficient outcome.

## 5. The Challenges of Online Classes

Even though online teaching and learning might seem to be effective and, in the era of COVID-19 pandemic and lockdown, probably the only possibility to continue the classes beyond the classrooms, this way of teaching raises several concerns and challenges as seen below in Table 1 [17,30,42,43,75,76,77,78,79,80,82,83]. The first disadvantage of online teaching that should be taken into consideration is the problem of reliable internet access and/or technology struggle that significantly affect the ability to participate in online learning. For instance, according to an OECD database, approximately 95% of students of such countries as Austria or Norway are provided with computers to learn at home, while in the case of Indonesia, the percentage is lowered only to 34%. This inequality raises doubts about whether proper teaching could be provided in the era of COVID-19 and if contact teaching could be completely replaced with online classes. Furthermore, even the students and teachers with more advanced technologies and better access to it might face such problems as difficulties with audio and/or video, login problems, and poor internet quality while having online classes, which significantly lower their quality and waste time aimed to provide knowledge [90]. Further, apart from classes, any exams or tests that needed to be performed via online platforms could raise doubts about students’ performance or even the possibility of falsifying the final results, as writing exams at home on computers could provide possibilities to cheat during exams [91]. Moreover, regarding anatomy education, the lack of possibilities to learn on cadavers and conduct dissections together by students significantly affects the learning outcomes [92]. Apart from educational aspects, the growing feelings of loneliness and isolation that further influence the academic scores and achievements should be taken into consideration [93]. While comparing the academic performance of students before and after the pandemic outbreak, a disturbing deterioration of the learning outcomes has been observed [94,95,96].

## 6. What does the Future Hold for Anatomy Education?

Apart from the challenges that relate to online education, the COVID-19 pandemic also deals with the future of anatomy students. Working with cadavers is considered a crucial part of anatomy education and contributes greatly to becoming a professional in the discipline, be that as a biomedical scientist, a dentist, or a medical doctor [97]. Clinical application of anatomical knowledge is essential to future physicians and connects the relevance of their education to their future training [13,98]. Thus, the student’s understanding of anatomy, concerning its clinical relevance, might suffer due to a lack of physical (practical teaching). It is therefore important for universities not to lose the focus on the clinical relevance while implementing the e-learning approach.

The low level of experience in dissection among current anatomy students might be a disadvantage [99]. Dissection of cadavers provides an ample chance for the acquisition of fine motor techniques in an environment free of stress, and this might have implications for the future of students and for the future of surgery more broadly [100].

Improper teaching of anatomy has been cited as one of the main reasons why students refuse to consider a surgical career, and although the present state of anatomy teaching is not deliberate, there is the possibility that students may receive a lower quality of teaching than before the pandemic [99,100,101]. Whether this will cause a reduction in applications to surgical training posts will be unknown for some time, but abandoning hurts the competency of potential surgeons.

## 7. Adapting Anatomy Education in the Post-COVID Era

During the COVID-19 pandemic, many medical schools and educational programs transitioned to remote learning, using various methods such as video conferencing, online presentations, instructional videos, and interactive 3D models. Some schools also utilized simulation programs to allow students hands-on experience with anatomy as well as increasing safety during the pandemic.

As the number of COVID-19 cases in Europe decreases, and restrictions are lifted, in-person classes have resumed at medical universities. During the lockdown, anatomy classes were solely conducted online using distance learning methods. The remaining question is whether we can use the experience gained from online classes to improve the quality of post-COVID-19 anatomy education?

New technologies are increasingly being used in anatomy education. Combining practical laboratory work with virtual methods has been found to be very beneficial. The majority of students surveyed preferred online lectures, as they found them to be more flexible and efficient [102]. They were able to customize their learning experience, have more time for self-study, easily access course materials and review them at their own pace. Many students found that studying in an environment of their choice helped in improving their concentration [103]. A survey by Potu et al. [104] found that most students preferred a mix of both in-person and online anatomy classes (57.4%) in the post-COVID-19 era.

Based on the data from Henri Schulte et al. [105], anatomy departments may offer shorter, more intensive, and active cadaver dissection sessions in small groups of 2–3 students more frequently instead of longer sessions with larger groups. These sessions should be complemented by online materials [105].

The use of technology such as virtual reality (VR) or augmented reality (AR) can also aid in anatomy education. AR, in particular, is associated with fewer side effects, such as nausea, than VR, and can be used for home learning as preparation for anatomy classes. Augmented reality is a technology that allows for the incorporation of digital elements into the real world. It can be used for teaching anatomy by allowing students to visualize and understand complex concepts related to the human body. AR can be used to visualize internal organs, body systems, and processes occurring within the body. Through interaction with 3D models, students can better understand complex relationships and dependencies between different body structures. AR can also enable students to conduct simulations and exercises, which can aid in understanding and retention of information [106].

It should be noted that access to cadavers and the ability to approach anatomy education in a hands-on manner is an essential element of teaching. Distance learning methods are a great supplement and aid for students, but they do not exist without in-person teaching. Ensuring access to cadavers and hands-on learning, while also utilizing technology and distance learning methods, can improve the quality of anatomy education in a post-COVID-19 world [107].

## 8. Conclusions

As education advances, so do the expectations of medical students when it comes to their learning approach. The teaching methodology of anatomy at medical universities has been evolving over the years. The COVID-19 pandemic has only accelerated the modernization and implementation of new solutions in the teaching of this subject. Teaching anatomy after the COVID-19 pandemic will certainly be different from what it was before. A rise in the usage of technology instruction methods has been seen. However, as the integration of new technologies inside the classroom is taking place, the importance of traditional teaching approaches that have a huge historic importance in training medical students, such as the dissection of cadavers, should not be dismissed and lose its value due to the rise of new pedagogies. Rather, the usage of new pedagogies can enhance the effectiveness of traditional teaching approaches such as cadaveric dissection. One great approach might be to combine several methods, ideally those that complement each other, as this will profit the students and would allow them to fully grasp ideas and concepts easier. An appropriate application of the latest technology with the use of traditional approaches to teaching can lead to enhancement in the understanding of human anatomy while ultimately improving the performance of students. Dissections and cadavers have remained the gold standard for a long period of time, even though they might be considered out-of-date, expensive, and time-consuming. With advances in technology, new teaching methodologies can help students understand and learn anatomy more easily and in a more efficient way.

## Figures and Tables

**Table 1:** Advantages and Disadvantages of distance learning.

Advantages	Disadvantages
Flexibility: students are able to study at home at any time of the day. Hence, there is no need for physical space, thus limiting the risk of infections (as in the COVID-19 pandemic).	Need for discipline: excessive flexibility may be disadvantageous to students who are not disciplined enough to meet the required activity without the physical presence of the tutor.
Content availability: content is always available for review, as the student has the option of downloading and saving the content.	Questions from students may not be addressed the very moment they arise, unlike in traditional classes.
Low cost compared to the prices of conventional classroom courses.	Loss in the quality of education, as the student does not have the opportunity to contribute actively in the preparation of lessons.
A single class video can serve several classes.	Feedback from the student takes longer. In regular classes, the tutor receives immediate feedback, but the same cannot be said of virtual classes. Thus, it takes longer for the teacher to be aware if the student has grasped the concept taught.

## Data Availability

Not applicable.
